# Relapse after Prolonged Remission in Philadelphia-Like Acute Lymphoblastic Leukemia

**DOI:** 10.1155/2019/3536517

**Published:** 2019-01-21

**Authors:** Gaurav Shah, Fady M. Mikhail, Andrew J. Carroll, Matthew Kutny, Nikolaos Papadantonakis

**Affiliations:** ^1^Internal Medicine, University of Alabama at Birmingham, Birmingham, AL, USA; ^2^Department of Genetics, University of Alabama at Birmingham, Birmingham, AL, USA; ^3^Division of Hematology/Oncology, Department of Pediatrics, University of Alabama at Birmingham, Birmingham, AL, USA; ^4^Division of Hematology/Oncology, Department of Medicine, University of Alabama at Birmingham, Birmingham, AL, USA

## Abstract

We describe a case of late relapse of Philadelphia-like acute lymphoblastic leukemia. The patient relapsed several years from diagnosis and responded to second salvage treatment. The case highlights the open questions regarding management of Philadelphia-like acute lymphoblastic leukemia.

## 1. Introduction

Recent advances identified a subgroup of Philadelphia (Ph) negative acute lymphoblastic leukemia (ALL) patients with dismal outcomes [1]. This group of patients has an expression profile similar to that of Ph positive ALL patients but lacks the characteristic *BCR-ABL1* gene fusion due to the translocation 9;22. This group encompasses a variety of kinase-activating lesions and is referred to as Ph-like ALL [2].

## 2. Case Description

We report the case of a 25-year-old young adult patient with relapsed Ph-like ALL at over 15 years from his initial ALL diagnosis at age 9. Patient's initial treatment included a multiagent chemotherapy regimen for standard risk (SR) pre-B ALL in accordance with the Pediatric Oncology Group (POG) 9905 regimen D [3]. Cytogenetics at initial diagnosis showed a balanced translocation t(3;12)(q21;q24), and *BCR-ABL1* gene fusion was negative. Flow cytometry revealed immature B-lineage phenotype. This initial diagnosis was during an era prior to the recognition of Ph-like ALL. The patient achieved first complete remission (CR) at end of induction therapy, and he completed the full regimen of chemotherapy for SR pre-B ALL. He remained in remission until 13 years off therapy (over 15 years from initial diagnosis) when he presented with a one-month history of night sweats, generalized weakness, petechiae, weight loss, lymphadenopathy, and splenomegaly.

Initial laboratory evaluation showed white blood count (WBC) of 44 × 10^3^/mm^3^ with a differential of 54% blast, hemoglobin of 8.3 g/dL, and platelet count of 24.6 × 10^3^/mm^3^. CT scans revealed scattered lymphadenopathy throughout chest, abdomen, and pelvis. The patient underwent a BM biopsy with an aspirate revealing 85% blasts. The bone marrow (BM) core biopsy had cellularity of 95% mostly composed by lymphoblasts. Flow cytometry on BM revealed atypical precursor B-cells with the following phenotype: CD19+, CD10hi, CD34 heterogenous, CD20+, CD9+, CD5−, and CD33− (see Table 1 for details). This phenotype was similar to his original diagnosis over 15 years prior. BM karyotype was 46,XY,t(3;12)(q21;q24) [20]. The same karyotype was present in previous samples, and later remission testing revealed that this translocation was constitutional. FISH analysis revealed that 86% of the cells had a rearrangement involving the *CRLF2* gene at Xp22.33/Yp11.32. BM metaphase FISH analysis demonstrated the presence of the t(X; 14)(p22.33;q32.3), which results in *IGH/CRLF2* fusion. BM FISH analysis was negative for the *BCR/ABL1* gene fusion, *MLL* gene rearrangement, and the *ETV6*/*RUNX1* gene fusion. Initial analysis revealed one copy of pathogenic variant in the kinase region of the *JAK2* gene (Nationwide Children's Hospital). Notably, subsequent molecular analysis (Neogenomics) at a later point on his treatment was positive for a fusion (*PAX5/ZCCHC7*) transcript involving *PAX5* and *ZCCHC7* genes resulting from t(9;9)(p13.2;p13.2) translocation. In addition, significantly high levels of *CRLF2* expression were detected. Moreover, mutations in *JAK2, CHD2, FIP1L1,* and *KDM6A* were detected. Of note, *JAK2* mutations are common among *CRLF2* rearranged cases [4]. The findings of *CRLF2* rearrangement with *JAK2* mutation are diagnostic of Ph-like ALL. Importantly, CSF assessment did not reveal presence of lymphoblasts.

The patient was treated with blinatumomab without significant complications. The BM biopsy after cycle 1 of blinatumomab revealed a reduction to the aspirate blasts to 41% while cellularity was decreased to 50–60% composed mostly by lymphoblasts. The peripheral WBC reduced to 7.51 × 10^3^/mm^3^ with 3% blasts. The hemoglobin improved to 11.4 g/dl and platelets to 158 × 10^3^/mm^3^. The patient then proceeded to the second cycle of blinatumomab, but within a few days, the WBC had increased from 5.84 to 108.6 × 10^3^/mm^3^ with 70% blasts.

Blinatumomab was discontinued, and after cytoreduction, the patient received inotuzumab ozogamicin (InO) at the recommended dose and schedule. Repeat BM biopsy after cycle 1 of InO showed no morphologic evidence of leukemia. Flow cytometry as well as FISH analysis for *CRLF2* rearrangement were negative (see table); karyotype was 46,XY,t(3;12)(q21;q24) [20]. The repeat CT scan showed improvement in diffuse lymphadenopathy and splenomegaly. The patient completed 2nd cycle without any complication, and another BM biopsy was performed again without morphological or flow cytometry evidence of ALL. Karyotype was again 46,XY,t(3;12)(q21;q24) [20], and therefore, it was considered to be most consistent with constitutional translocation.

Following further imaging studies, allogeneic bone marrow transplant (AlloHSCT) was pursued. Conditioning regimen was based on total body irradiation and cyclophosphamide while the donor was matched unrelated. The patient developed infectious complications, electrolyte abnormalities, and arrhythmias during his AlloHSCT course and expired. There was no evidence of veno-occlusive disease at the time of death.

## 3. Discussion

Children, adolescents, and adults with Ph-like ALL have a dismal prognosis [2, 5]. The use of dasatinib or ruxolitinib with chemotherapy in this subgroup of patients is underway (ClinicalTrials.gov Identifier: NCT02420717) in an attempt to improve outcomes [6].

Patients in CR remain at risk of relapse; the median EFS was 17.2 months for adult patients treated on the study of Jain et al. [7]. In a recent report of pediatric patients, the median relapse free survival was less than 3 years with a patient relapsing 10.86 years after diagnosis [8].

The Ph-like ALL patient described in this case report relapsed 15 years after initial diagnosis. Our case is one of the very late relapses in ALL reported in the literature and further highlights that such late relapses can occur in Ph-like ALL patients. The mechanisms that can lead to such a late relapse are not well defined (discussed in Norkin et al.) [9]. One hypothesis is that a leukemic clone persists in a semidormant state kept in check by the immune system but then escapes immune system surveillance many years later. Alternatively, a quiescent leukemic stem cell can survive the course of initial treatments and later through further mutational events attain a proliferative state that becomes clinically apparent.

The optimal management of Ph-like ALL patients that relapse remains uncertain. Although the sensitivity of Ph-like ALL to therapy with kinase inhibitors (including the JAK2 inhibitor ruxolitinib and the ABL1 kinase inhibitor dasatinib) has been reported, many aspects of the optimal use of these agents remain unclear. Moreover, the safety of the combination of dasatinib or ruxolitinib with immunotherapies (including the immunoconjugate InO and the bispecific T-cell engaging antibody Blinatumomab) that are FDA approved for relapsed ALL has not been extensively studied [10].

We utilized blinatumomab as salvage regimen as it has been associated with favorable outcomes in relapsed/refractory ALL compared with conventional chemotherapy [11]. Our patient did not respond to blinatumomab. We next attempted treatment with InO as it is also approved for relapsed/refractory ALL [12]. Moreover, in a case series after treatment failure with blinatumomab, the InO was able to achieve 75% response [13]. In this single institution experience, 7 of 12 patients who achieved response received InO in combination with chemotherapy. Our patient responded to InO and was able to proceed to AlloHSCT.

Notably, we were not able to find guidance in the literature regarding the optimal management of relapsed Ph-like ALL patients. Studies focusing on the outcomes of salvage treatments in the Ph-like ALL would be very helpful to inform a preferred approach. For example, the association of specific molecular aberrations in Ph-like ALL patients with FDA approved and NCCN category 1 recommended treatments such as blinatumomab or InO would be of particular interest.

## 4. Conclusion

Our case of very late relapse of a Ph-like ALL patient highlights that although uncommon, late occurrence can happen. Moreover, optimal salvage regimen for Ph-like ALL is not defined, and further studies are eagerly awaited.

## Figures and Tables

**Table 1:** Summary of hematological and flow cytometry findings, molecular aberrations, and cytogenetics at various stages of clinical course.

	Relapse	Postcycle 1 blinatumomab	Blinatumomab treatment failure	Postcycle 1 inotuzumab ozogamicin	Prior to AlloHSCT
WBC in 10^3^/mm^3^ (blast percentage)	44 (54%)	7.51 (5%)	108 (79%)	7.45 (0%)	6.45 (0%)
Platelet count (10^3^/mm^3^)	24.6	158	112	194	167
Flow cytometry	Precursor B-ALL (76% blast)	Precursor B-ALL (32% blast)	Precursor B-ALL (79% blast)	No evidence of ALL	No evidence of ALL
Bone marrow diagnosis	Aspirate: 85% blasts	Aspirate: 41% blasts	Not performed	Aspirate/core: no morphologic or phenotypic evidence of B-ALL	Aspirate/core: no morphologic or phenotypic evidence of B-ALL
Pertinent CD markers on bone marrow (peripheral blood at blinatumomab treatment failure)	CD19+, CD10 high, CD45 low, CD34±, CD20+, zCD9+, CD38+, CD58+, sIg−, CD5−, CD22+, CD13−/low, and CD33−	CD19+, CD10 high, CD45 low, CD34±, CD20+, CD22+, CD38+, CD58+, sIg−, CD13−, and CD33−	CD19+, CD20+, CD10hi, CD45lo, CD34 heterogeneous, CD117−, CD22+, CD38+, CD58lo, CD9+, surface Ig−, CD13−, and CD33−		
CRLF2 rearrangement at Xp22.33/Yp11.32	86% of cells positive	40% of cell positive	76% of cell positive	Negative	Negative
Karyotype	46,XY,t(3;12)(q21;q24) [20]	46,XY,t(3; 12)(q21;q24.1) [20]	46,XY,t(3; 12)(q21;q24.1) [20]	46,XY,t(3; 12)(q21;q24.1)c [20]	46,XY,t(3; 12)(q21;q24.1)c [20]
Pertinent molecular genetics	1 copy of the pathogenic variant in the kinase region of the *JAK2* gene		Fusion of PAX5-ZCCHC7. Mutation in JAK2, CHD2, FIP1L1, and KDM6A		
